# Separator Skim-milk

**Published:** 1895-05

**Authors:** 


Prof. C. B. Cochran, Inspector of Food of the State Board of Agriculture, recently addressed the Milk Symposium under the auspices of the Pennsylvania Horticultural Society, in which he said: "No mammal is strictly herbivorous. Even if the adult animal derives its sole nourishment from plants, the younger feeds on milk, a secretion furnished by nature as food for the young. The typical food of the adult cow is grass, but of the calf is milk. The general fact that the food of the adult differs from that of the young holds true in all other mammals, including man. A very plausible reason for this seems to suggest itself when we study the composition of the animal body and the nourishing constituents of our foods. The food of animals can be divided into four classes, and in one or the other of these four classes every substance forming a food for man or the lower animals can be classed. These four classes are the mineral constituents, the hydrocarbons, the carbohydrates and the proteids. The carbohydrates belong almost exclusively to the plant kingdom. They can scarcely be said to enter at all into the formation of the animal organism, but, on the other hand, they are the sole building material of plants. Cellulose, starch and sugar are the important members of this group.

"The proteids, on the other hand, are the building materials of animals. The chemical composition of the tissues of plants and animals we thus see are different. The proteids and the carbohydrates differ decidedly in all their physical and chemical properties. Plants make and use proteids. The life of the plant depends upon its ability to make proteids, but it does not use this valuable material to construct its body. Animals use carbohydrates. Three-fifths to four-fifths of the animal's food are carbohydrates. These substances seem to be, in many cases at least, essential to the life of the animal. Yet the animal organism is not composed of carbohydrates. Nor can it build up its organs out of these materials. To build up its proteid tissues it must have proteids. The animal is unable to make proteids for itself. It must get them from plants. But plant materials are not rich in proteids.

"Milk, on the other hand, is rich in this class of food materials, and furnishes to the newborn mammal, in an easily digestible and assimilable condition, the proteid material to meet the demand that now exists for rapid tissue formation. For this reason, and also because the young mammal does not possess the power of readily digesting the vegetable carbohydrates, nature endows the female parent with the power of secreting a specially prepared food.

"The chief use of the carbohydrates to the animal seems to be to serve as fuel. By their oxidation in the body of the animal heat and energy are maintained. The proteids are not well adapted to this latter purpose, partly, no doubt, because of the difficulty of eliminating the products of their oxidation. In the early life of the mammal the chief use of food is for tissue formation. At a later period the principal demand for food is for fuel purposes. The necessity for tissue formation still exists, but to a more limited extent. For this reason, and also because of the only partially developed digestive powers a difference necessarily exists in the character of the food.

"Without going into details, I may say that experience has shown that the above list of alimentary principles should exist in our foods in certain definite proportions. A man engaged in moderate labor should have per day: Carbohydrates, 15 ounces; hydrocarbons, 4.5; proteids, 4.5; mineral matters, 0.75, giving, water free, total solids, 24.75 ounces.

"Since our solid foods are about 50 per cent. water, we must double these figures in order to obtain the actual amount taken daily, thus obtaining about 48 ounces actual food consumed per day.

"The composition of cow's milk is about as follows: Mineral matter, 0.72 per cent.; proteids, 3.6; carbohydrates, 4.4; hydrocarbons, 3.9; total solids, 12.62 per cent.

"In spite of all that has been said against it by members of the Philadelphia Board of Health and others, the fact still remains that separator skim milk is a wholesome and valuable article of food. In a letter from Prof. Woll he speaks very decidedly in favor of the use of separator milk. In my opinion, the only excuse that the Philadelphia Board of Health can offer for the position they have taken in reference to separator milk is that they feel obliged to adopt extreme measures in order to prevent fraud in the sale of skim milk for full milk. Separator milk and cream have far better keeping qualities than the milk from which they were obtained. Practically every trace of dirt and the greater part of the micro-organisms contained in the milk are removed by the centrifugal process and retained in the slime collected in the separator. Separator skim milk possesses every advantage over the old-fashioned hand-skimmed milk, and its use should be encouraged. Outside of the large cities separator skim milk is not easily obtained. In many towns little or no effort is made to sell it. The result is that people do not learn to use it, and hence cannot become acquainted with its food value.

"Skim milk contains about 9 per cent. of solids. When the water is evaporated from these solids a valuable food material remains, which consists of all the proteids, carbohydrates and mineral matter of the milk. The attempts thus far made to bring this material into use either as a food for man or the lower animals have met with very little success. Yet, when ground into a meal it can be mixed with boiled potatoes and made into potato cakes. I have several times experimented in this way with the dried solids of separator skim milk, and I find that it can be mixed with vegetable food materials and thus be made to supply a rational diet at small expense. Three years ago I exhibited potato cakes thus made at a Farmers' Institute. As one of the substances used in the dietary of armies and government expeditions, and for the inmates of charitable and penal institutions, the dry residue of skim milk is worthy of a place, or at least a trial. I see no reason why they cannot be combined with any farinaceous food, such as rice, corn meal, rye or wheat flour, in the preparation of a compact, nutritious food.

"When skim milk is used in fattening pigs the animals should also receive a fair allowance of carbohydrate food. To obtain the best results for the food fed the proteid material in the food of pigs should form about one-seventh of the digestible organic solids, but in the solids of skim milk the proteid material forms about one-half of the organic matter.

"The day will soon have passed when the people of this country can afford to waste their food supply. The value of the proteids or tissue-forming foods, especially those obtained from the animal kingdom, will increase as our population increases. Fifty years has seen a phenomenally rapid growth in all of the country west of the Mississippi River. If this growth continues at anything like the same rate, the vast areas now devoted to cattle grazing will be greatly lessened. As our country increases in population it is almost certain that our production of meat will not only fail to increase correspondingly, but will actually decrease. Meats will therefore form a smaller part of our diet. Even now, men who have families and are working on low wages cannot afford to buy meats for daily use. Extravagance in foods is one of the causes of poverty in every city and town. The cost of a food bears no relation whatever to its value as a nourishment to the healthy human body. Skim milk contains animal proteids in a form easily digested and assimilated. It is undoubtedly one of the cheapest, if not the very cheapest, wholesome food of animal origin which can be supplied in immense quantities to all the markets of our country, and to learn to utilize this product of the dairy I regard as a problem of national importance."

